# Dose-Response Effect and Dose-Toxicity in Stereotactic Radiotherapy for Brain Metastases: A Review

**DOI:** 10.3390/cancers13236086

**Published:** 2021-12-02

**Authors:** Maxime Loo, Jean-Baptiste Clavier, Justine Attal Khalifa, Elisabeth Moyal, Jonathan Khalifa

**Affiliations:** 1 Radiotherapy Department, University Cancer Institute of Toulouse—Oncopôle, 31100 Toulouse, France; attalkhalifa.justine@iuct-oncopole.fr (J.A.K.); moyal.elizabeth@iuct-oncopole.fr (E.M.); Khalifa.Jonathan@iuct-oncopole.fr (J.K.); 2 Radiotherapy Department, Strasbourg Europe Cancer Institute (ICANS), 67033 Strasbourg, France; jb.clavier@icans.eu

**Keywords:** stereotactic radiotherapy, radiosurgery, brain metastases, fractionation, dose-effect relation

## Abstract

**Simple Summary:**

Brain metastases are one of the most frequent complications for cancer patients. Stereotactic radiosurgery is considered a cornerstone treatment for patients with limited brain metastases and the ideal dose and fractionation schedule still remain unknown. The aim of this literature review is to discuss the dose-effect relation in brain metastases treated by stereotactic radiosurgery, accounting for fractionation and technical considerations.

**Abstract:**

For more than two decades, stereotactic radiosurgery has been considered a cornerstone treatment for patients with limited brain metastases. Historically, radiosurgery in a single fraction has been the standard of care but recent technical advances have also enabled the delivery of hypofractionated stereotactic radiotherapy for dedicated situations. Only few studies have investigated the efficacy and toxicity profile of different hypofractionated schedules but, to date, the ideal dose and fractionation schedule still remains unknown. Moreover, the linear-quadratic model is being debated regarding high dose per fraction. Recent studies shown the radiation schedule is a critical factor in the immunomodulatory responses. The aim of this literature review was to discuss the dose–effect relation in brain metastases treated by stereotactic radiosurgery accounting for fractionation and technical considerations. Efficacy and toxicity data were analyzed in the light of recent published data. Only retrospective and heterogeneous data were available. We attempted to present the relevant data with caution. A ${\mathrm{BED}}_{10}$ of 40 to 50 Gy seems associated with a 12-month local control rate >70%. A ${\mathrm{BED}}_{10}$ of 50 to 60 Gy seems to achieve a 12-month local control rate at least of 80% at 12 months. In the brain metastases radiosurgery series, for single-fraction schedule, a V12 Gy < 5 to 10 cc was associated to 7.1–22.5% radionecrosis rate. For three-fractions schedule, V18 Gy < 26–30 cc, V21 Gy < 21 cc and V23 Gy < 5–7 cc were associated with about 0–14% radionecrosis rate. For five-fractions schedule, V30 Gy < 10–30 cc, V 28.8 Gy < 3–7 cc and V25 Gy < 16 cc were associated with about 2–14% symptomatic radionecrosis rate. There are still no prospective trials comparing radiosurgery to fractionated stereotactic irradiation.

## 1. Introduction

Brain metastases are one of the most frequent complications for cancer patients and occur in 20 to 40% of them [1]. Due to the low response rate of brain metastases to chemotherapy [2], whole-brain radiotherapy (WBRT) has long been considered the cornerstone of treatment, providing palliation of symptoms in 70% and an intracranial response in 50% [3]. However, WBRT is responsible for neurocognitive decline, thus, stereotactic radiotherapy (SRT) has emerged as an alternative to WBRT in patients with a limited number of lesions [4,5,6].

Several randomized trials demonstrated that SRT or surgery associated with WBRT improved local control compared to WBRT alone [7]. Moreover, overall survival (OS) was better with the addition of surgery or SRT to WBRT in patients with a single brain metastasis [8]. WBRT plus SRT or surgery increases control within the brain compared to surgery or SRT alone, but it does not improve OS [8,9,10,11,12]. However, the association may lead to a greater neurocognitive decline [13,14], especially in long-term survivors. New systemic therapies such as targeted therapies and immunotherapy are challenging these strategies. For example, in patients with non-small-cell lung cancer (NSCLC), a dramatic increase of intracranial response rate was observed with tyrosine kinase inhibitors (TKIs) in epidermal growth factor rate (EGFR)-mutated patients [15,16].

SRT alone has high efficacy with more than 75% local control at 1 year in the most recent series [17,18,19] and low toxicity, so it has become a cornerstone treatment for patients with limited brain metastases.

Accessibility to SRT has improved considerably in recent years with significant advances in precision, reproducibility and patient comfort. The feasibility of frameless treatment has led to the delivery of hypofractionated SRT, usually with three to five fractions. The delivery of the dose is closely linked to the device used for treatment. Historically, radiosurgery described in the RTOG trial 90-05 [20] was the standard of treatment. However, from a radiobiological point of view, fractionation allows an increase in the biologically effective dose while sparing healthy tissue. In the case of large tumors or in the vicinity of sensitive organs, fractionated treatment has a higher efficacy and lower toxicity compared to a single fraction [17,21]. Large tumors may be more radioresistant due to hypoxia in some areas of them. Since the ideal dose and fractionation schedule remain largely unknown, this review explored the dose–response relationship in the treatment of brain metastases.

## 2. Materials and Methods

This narrative critical review was conducted using MEDLINE via PubMed by identifying articles regarding SRT of brain metastases. Searches for original and review articles were conducted by ML and JK from January 1990 until January 2021. The search was limited to articles written in English; unpublished material and abstracts were excluded from this review. General search terms (including both Medical Subject Headings (MeSH) and free text words) included the following: “stereotactic radiosurgery”, “brain metastases”, “stereotactic radiotherapy”, “hypofractionated radiosurgery”, “fractionated radiotherapy”. The bibliographic references of the selected articles were also reviewed and included according to their relevance.

This narrative review first aimed at clarifying general considerations regarding technical aspects of SRT for brain metastases, before focusing on the dose–efficacy relation, fractionation and the dose–toxicity relation. To do so, only relevant references were selected within the indicated period and analyzed. A special focus was placed on the comparison of single-fraction vs. multiple fractions of SRT in brain metastases with selection of only articles providing data on metastases volumes. Resected brain metastases treated by SRT were excluded except in the dose–toxicity analysis.

## 3. Technical Considerations

### 3.1. Treatment Devices

Currently, a wide variety of systems enable the delivery of SRT: Cobalt-based systems such as GammaKnife (GK), robotic linear accelerator such as CyberKnife (Accuray, Sunnyvale) and dedicated linear accelerator with gantry (LINAC) such as Novalis TrueBeam STX (Varian, Palo Alto, CA, USA), VersaHD (Elekta, Stockholm, Sweden) and Vero (Mitsubishi, Tokyo, Japan).

The dosimetric properties of these devices vary and could theoretically influence the effectiveness and tolerance of irradiation. Several comparative studies have found contradictory dosimetric results. Sio et al. [22] showed that treatment plans for brain metastases on GK or CyberKnife were globally comparable for target coverage and minimum dose coverage, but the CyberKnife had a better conformity index and a trend to irradiate more volume of normal brain tissue receiving 12 Gy ($V_{12}$) (not significant). Wowra et al. [23] found a lower dose gradient and a lower $V_{10}$ with the CyberKnife than with the GK for similar clinical results (secondary criteria). Ma et al. [24] reported less irradiation of normal tissue with the GK than with the CyberKnife or Novalis with a discretely lower $V_{12}$ (3.5 cc vs. about 5 cc for three given targets). Treuer et al. [25] compared treatment plans with the CyberKnife vs. LINAC in stereotactic radiosurgery (SRS) on 23 target lesions and reported slightly better coverage, conformity, and mean minimum dose with the CyberKnife but with a same $V_{10}$. It should be noted that treatment with GK takes longer than with LINAC, especially when there are more targets.

Although the RTOG 90-05 study [20] showed a higher propensity for local relapse in patients treated with LINAC vs. GK (RR of 2.84, *p* = 0.018) and the dosimetric properties of the devices are somewhat different, the type of machine used for treatment does not appear to influence efficacy. The local control rates of published SRS series on LINAC or GK are similar [26]. The authors found no differences in the RTOG 95-08 phase III trial [8], nor in another trial [27] that analyzed an SRS boost in different modalities after whole-brain irradiation. This multi-institutional analysis of 502 patients also showed a similar efficacy of the SRS boost regardless of the treatment device used.

A recent randomized phase III trial [28] compared SRS irradiation in GK and LINAC in 168 patients with 292 brain metastases. The prescriptions were 20–24 Gy on the 50% for GK and 24 Gy on the PTV marginal isodose for LINAC. The primary endpoint was the incidence of radionecrosis. The rate of grade III radionecrosis was higher in the GK group (1% vs. 0%) and the local control rate at 1 year was 98.8% and 96.2% for the SRS and LINAC groups, respectively (*p* = 0.96). Another recent retrospective study [29] also paradoxically reported more radionecrosis with GK than with LINAC (HR = 4.42; *p* = 0.019) in patients with two brain metastases treated in a single fraction, despite a margin of 2 to 3 mm with LINAC vs. 0 on the GK and a much lower V12 for the latter. 

Nath et al. [30] also noted that local control and overall survival rates were similar when examining SRS series with invasive and non-invasive frames (either LINAC or GK). The same was true for benign tumors [31,32]. Other authors [23,33] also reported the same efficacy (secondary endpoint) in patients treated with GK or CyberKnife in their dosimetry studies.

Additionally, there are different treatment planning approaches. The new HyperArc VMAT (volumetric modulated arctherapy) planning is promising with a higher conformity and rapid dose falloff compared to conventional VMAT [34,35]. 

### 3.2. Physical Factors

While the effect of irradiation depends on the dose, physical factors can also influence the biological response such as duration of dose delivery, time between two fractions, and overall treatment time.

Increasing the duration of irradiation administration during the same fraction may reduce efficacy because it allows the repair of sub-lethal cellular events during the fraction [36], especially for tissues with a low α/β ratio. This is particularly the case with the GK when the cobalt sources are at the end of their life cycle. On the contrary, the FFF (filter flattening free) mode available on some LINACs enables an increase in the dose rate and a reduction in the duration of the session. Increasing the time between fractions or overall treatment time also reduces efficacy by allowing repopulation, although a sufficient interval (24 h) between two fractions allows reoxygenation of the tissue and may increase efficacy.

Owing to a large penumbra, one of the physical characteristics of the irradiation of small fields is the inhomogeneity of the beam and the dose delivered, with a steep dose gradient between the isocenter and the periphery. A steep dose gradient probably leads to better therapeutic efficacy [37], an additional effect that is potentially underestimated by the linear-quadratic model [38]. 

### 3.3. Target Volume and Prescription

The definition of the irradiation volumes requires a CT scan and a localization MRI fusion with thin sections (at least 2 mm for a field strength of at least 1.5 T), T1-weighted sequences that are injected, and T2 FLAIR [39]. The time between imaging and the start of treatment should be as short as possible so as not to underestimate the tumor volume. The macroscopic target volume (GTV) is defined by contrast enhancement on MRI or CT (if injected). The clinical target volume (CTV) is defined by adding a margin of 0 or 1 mm to the GTV, because microscopic invasion of healthy tissue of a metastatic brain lesion does not exceed 1 mm [40], except in small cell carcinomas and melanomas. The planned target volume (PTV) depends on the treatment technique used, usually GTV = CTV = PTV for radiosurgery with invasive fixation (e.g., GK). The size of the PTV margin depends on the treatment and immobilization techniques used, usually varying from 0 to 3 mm. There is currently no consensus on the definition of PTV margins due to the wide range of available techniques. To increase the peripheral dose, one can directly increase the prescribed dose on the reference isodose or increase the PTV margin. Indeed, in one study [41], adding a margin of 2 mm vs. 0 mm to the GTV to define PTV in patients treated with LINAC-SRS increased the rate of complications without increasing local control. In another study [42], the addition of a 1 mm margin to the GTV to define PTV increased local control without increasing the rate of complications. Kirkpatrick et al. [43] showed in a randomized study that a 3 mm margin added to the GTV to define PTV was associated with a higher rate of radionecrosis compared to a 1 or 2 mm margin, for similar local control, in patients treated with SRS on LINAC with a thermoformed mask. The inhomogeneity of the dose is associated with a potentially better efficacy due to a possible over-impression of the dose within the target in hypoxic areas, which would confer an advantage for so-called radioresistant tumors. Recently, a study by Lucia et al. [37] reported better efficacy with inhomogeneous doses (same marginal isodose and dose variable at isocenter) with a local control at 1 year of 93% vs. 78% for the same homogeneous dose with LINAC (*p* = 0.005). Finally, it should be remembered that from one treatment planning system (TPS) to another, the use of a different dose calculation algorithm can modify the dose by more than 15% [44]. 

## 4. Dose-Effect Relation

### 4.1. Dose-Efficacy Relation

The RTOG 90-05 [20] dose escalation trial gave the main radiation schedules used in radiosurgery, although the relationship between dose and local control was not reported. The study established schedules of 24 Gy, 18 Gy and 15 Gy for tumors of size smaller than or equal to 20 mm, 21–30 mm and 31–40 mm, respectively. The use of lower doses for larger volumes to spare the volume of normal tissue irradiated may lead to poorer local control, while the increase in dose results in a higher rate of radionecrosis. Another retrospective study [45] of 100 patients with 219 lesions treated with radiosurgery showed better local control with a dose of 18 Gy or greater with 93% and 90% local control at 6 and 12 months, respectively, compared to patients treated with a lower dose (*p* = 0.0001).

In 2011, Wiggenraad et al. [26] conducted the first systematic review of the dose–response relation in the stereotactic irradiation of brain metastases. They selected 11 studies [19,46,47,48,49,50,51,52,53,54,55] from 1990 to 2009, of which all but one (prospective phase II trial) were retrospective. The number of patients ranged from 20 to 202 depending on the studies, all histologies combined, for a number of metastases ranging from 20 to 375. Seven studies used a single-fraction regimen. Three of the 11 used the GK and 8 used a LINAC. Four studies used the RTOG 90-05 dose regimen but with variable prescription isodoses ranging from 50 to 100%. The margin from GTV to PTV ranged from 0 to 3 mm. Five studies included patients irradiated with brain in toto (ranging from 8% to 76%). Tumor diameter did not exceed 5 cm and for most studies it was ≤4 cm.

Local control at 6 and 12 months in the single-fraction studies ranged from 82% to 100% and 37% to 93%, respectively. Local control at 6 and 12 months in multi-fraction studies ranged from 89 to 100% and from 70 to 89%, respectively. Local control at 6 months was greater than 80% in all but one series. In the single-fraction series, local control at 12 months was greater than 80% for doses above 20 Gy and greater than 60% for doses above 18 Gy.

To achieve 70% or more local control at 12 months, they found that a ${\mathrm{BED}}_{12}$ (biological effective dose with an alpha/beta of 12 Gy) of at least 40 Gy was required, which is equivalent to a single fraction of 20 Gy or three fractions of 8.5 Gy. Factors such as tumor volume, prescribing isodose, GTV-PTV margins and tumor histology could not be analyzed in this study. However, the authors could not exclude that the better local control observed in hypofractionated series was not only due to a higher BED but also to higher GTV-PTV margins compared to single fraction series, generally equal to 0 mm.

In a systematic review of the literature on brain metastases treated with hypofractionated SRT, Rodrigues et al. [56] reported local control at 6 months ranging from 67 to 97%, at 1 year from 59 to 91% and at 2 years from 45 to 83%, for ${\mathrm{BED}}_{10}$ ranging from 24 to 100.8 Gy in the series excluding previous whole-brain irradiation [19,52,55,57,58,59,60,61,62,63,64,65,66,67,68,69,70,71,72,73,74,75,76]. All series combined, a direct correlation was observed between ${\mathrm{BED}}_{10}$ and local control, with a Pearson score of 0.57 for local control at 6 months and 0.62 for local control at 12 months. A total of 36 retrospective studies were included, 23 of which excluded previous whole-brain irradiation. WBRT may be a confounding factor but one study [77] did not find any differences in LC or OS between SRS alone vs. WBRT associated to SRS in 223 patients and 360 brain metastases.

A more recent systematic review [78] including 10 articles [50,52,55,57,58,64,79,80,81,82] with 720 brain metastases treated with hypofractionated SRT (HFSRT) showed that the tumor control probability (TCP) increases with the ${\mathrm{BED}}_{10}$ with a hazard ratio (HR) equal to 0.77 for every 10 ${\mathrm{Gy}}_{10}$ with *p* = 0.009. Local control at 1 and 2 years was 84% and 73%, respectively, for lesions treated with a ${\mathrm{BED}}_{10}$ of at least 57.6 Gy. For those treated with BEDs less than 57.6 Gy, local control at 1 and 2 years was 72% and 60%. All treatments were performed on LINAC with prescription isodoses ranging from 70 to 100%. 

An interesting approach reported by Matsuyama et al. [83] is to prescribe doses in BED regardless of the fractionation schedule used or the tumor size. They delivered an effective biological dose of approximately 80 Gy (α/β = 10) in HFSRT for almost all 573 secondary brain metastases from NSCLC in 299 patients. They reported a high local control at 6 and 12 months of 96.3% and 94.5%, respectively. In total, a 12-month local control rate >70% seems to be achieved with a ${\mathrm{BED}}_{10}$ of 40 to 50 Gy. A ${\mathrm{BED}}_{10}$ of 50 to 60 Gy seems to achieve a 12-month local control rate of at least 80% at 12 months.

Factors other than dose may also influence the response to irradiation such as histology [18,84,85] with more or less radioresistant tumor cells (melanoma) or systemic treatments [86,87,88,89] (targeted therapies or immunotherapy).

### 4.2. Single or Multiple Fractions?

There are currently no prospective trials comparing HFSRT with radiosurgery, and comparative retrospective studies are also rare (see Table 1). Among them, Kim et al. [64] reported 58 patients treated with SRS vs. 40 with HFSRT on LINAC with an isotropic margin of 1 mm for PTV, median doses of 20 Gy (15–22 Gy) for radiosurgery, 36 Gy (30–42 Gy) in 6 fractions for HFSRT, and similar prescription isodoses (90 and 91%, respectively). They found a local relapse-free survival at 6 months of 81% vs. 97% in the group of patients treated with SRS vs. HFSRT, and at 1 year, 71% vs. 69% *p* = 0.31, respectively. The median ${\mathrm{BED}}_{10}$ was 60 Gy for SRS and 56 Gy for HFSRT. It should be noted that 40% of patients in the HFSRT group had whole-brain irradiation compared to 21% in the SRS group. PTV volumes were higher in the HFSRT vs. SRS group with a median volume of 5.00 mL vs. 2.21, respectively. Wiggenraad et al. [90] did not find any differences in LC in large brain metastases between 15 Gy in a single fraction vs. 24 Gy in three fractions in 92 patients treated by SRT with globally similar metastasis volumes but 15% and 25% of patients received previous WB in HFSRT and SRS group, respectively. The ${\mathrm{BED}}_{10}$ was similar. Fokas et al. [91] compared three dose regimens: SRS according to RTOG 90-05 doses, HFSRT 7 × 5 Gy and 10 × 4 Gy. A total of 260 patients with 1 to 3 metastases were treated on LINAC with an isotropic margin added to the GTV of 2 mm in the SRS group and 3 mm in the HFSRT groups. They reported local relapse-free survival at 6 months and 1 year of 84% and 73%, 87% and 75%, 81% and 71%, respectively, for each group (*p* = 0.191). The median PTV volume was 1.87 (0.03–11.17) for the SRS group, 2.04 (1.17–18.71) in the 7 × 5 Gy group and 5.93 in the 10 × 4 Gy group (2.7–23.16). The calculated BEDs were 60 ${\mathrm{Gy}}_{10}$ and 153 ${\mathrm{Gy}}_{3}$, 52.5 ${\mathrm{Gy}}_{10}$ and 93.3 ${\mathrm{Gy}}_{3}$, 56 ${\mathrm{Gy}}_{10}$ and 92 ${\mathrm{Gy}}_{3}$ in the three groups, respectively. Ishihara et al. [18] compared 53 patients with 214 secondary brain lesions, 138 in the SRS group and 76 in the HFSRT group. Treatment doses were 15 to 25 Gy for SRS with a median marginal dose of 20 Gy (median ${\mathrm{BED}}_{10}$ 60 Gy), 35 Gy in 5 fractions with a median marginal dose of 28 Gy (median ${\mathrm{BED}}_{10}$ 43.7 Gy) for HFSRT with LINAC, prescription isodoses ranging from 80 to 90% and isotropic margins added to the GTV to define PTV ranging from 1 to 2 mm for HFSRT and 1 mm for SRS. They reported a 1-year local control of 83.6% in the HFSRT group. For PTV volumes greater than 4 cc, local control at 1 year was 46.2% vs. 80.6% for ${\mathrm{BED}}_{10}$ < 51 Gy vs. ${\mathrm{BED}}_{10}$ ≥ 51 Gy (*p* = 0.024). No difference in local control was found between the two groups (HFSRT vs. SRS) for PTV volumes less than 4 cc (94.4% vs. 99.2% *p* = 0.195). The median PTV volume was 6.2 cc (0.1–29.5 cc) for HFSRT and 0.7 cc (0.1–8.3 cc) for SRS. Minniti et al. [17] compared 289 patients with 343 metastases: 151 received SRS treatment and 138 received HFSRT. Lesion diameters were all greater than 2 cm. Local control at 6 and 12 months was 94% vs. 97% and 77% vs. 90% (*p* = 0.01) in the SRS vs. HFSRT group, respectively. The difference in local control at 1 year in favor of HFSRT remained after a propensity score achieved in 208 patients (91% vs. 76%, *p* = 0.01) by matching on age, sex, histology, tumor size, and irradiated volumes. The doses used in the SRS group were 18 Gy for lesions between 2 and 3 cm and 15–16 Gy for lesions greater than 3 cm. The dose used in the HFSRT group was 27 Gy in 3 fractions (${\mathrm{BED}}_{12}$ = 40 Gy, equivalent to a single fraction of about 22 Gy). The prescription was made on 80 to 90% isodose lines. All patients were treated on LINAC with an isotropic margin added to the GTV of 2 mm (50% of patients) or 1 mm to define PTV. The median PTV volume was 12.2 (4.4–32) for SRS and 17.9 (5.6–54) for HFSRT. Feuvret et al. [82] compared 36 patients with large secondary brain lesions >3 cm, 24 by SRS (14 Gy on the 70% isodose line) and 12 in HFSRT (3 fractions of 7.7 Gy daily on the 70% isodose line), with a median GTV volume of 15.69 cc (diameter 3.8 cm), and 29.4 cc (equivalent diameter of 4.5 cm), respectively. They reported local control at 12 and 24 months of 100% and 64% in the HFSRT group vs. 58% and 48% in the SRS group (*p* = 0.06). However, although all patients were treated on LINAC, the margins added to the GTV to create the PTV were 1 mm in the SRS group and 2 mm in the HFSRT group. The BED was lower for the SRS regimen, with a ${\mathrm{BED}}_{10}$ = 33.6 Gy vs. 41 Gy for HFSRT. Chon et al. [92] showed better efficacy and tolerance with a hypofractionated regimen treated with CyberKnife (median 35 Gy, 3 to 5 fractions) in 100 patients with brain metastases of 2.5 to 3 cm compared to a single dose treated with GK (median 20 Gy) with a local control at 1 year of 92.4% vs. 66.6%, respectively (*p* = 0.028). Loo et al. [93] reported similar efficacy in 152 patients with 246 brain lesions on the same LINAC and same margins between a single dose of 14 Gy prescribed on the 70% isodose line vs. 23.31 Gy in 3 fractions on the 70% isodose line with a local control at 1 year of 88.1% vs. 78.4% (*p* = 0.06). Interestingly, the tumor volume was statistically higher in the HFSRT group (median 0.21 cc vs. median 2.36 cc for SRS vs. HFSRT), although a subgroup size analysis showed no differences between both fractionations. A study [94] looking only at brain metastases of radioresistant histological origin (kidney and melanoma) collecting 193 lesions <3 cm treated with CyberKnife also found no difference in local control (*p* = 0.38) in SRS (median of 20 Gy) vs. HFSRT (median of 10 Gy/fraction), but also with a large difference in treated volumes (0.47 cc vs. 1.75 cc for median PTV). Remick et al. [95], in a multicenter study of 156 patients with 335 brain metastases, found no difference in local control at 1 year (91% in SRS vs. 85% in HFSRT, *p* = 0.26). Lesions were treated on different devices with variable margins, one-third of patients had previously received WBRT and tumor volumes were lower in the SRS group. They reported better efficacy in multivariate analysis for HFSRT regimens with a $BED_{10}$ ≥ 50 Gy (*p* = 0.09). In study of 120 patients with 190 metastases, Putz et al. [96] found a better LC and a lower RN with fractionated SRT vs. SRS with a 12-month LC at 70.2% vs. 55.6%. Prior WB regarded about 20% of patients in each group and the median metastasis volume was much higher in the HFSRT group but LC was also improved in HFSRT in the <1 or >1 cm metastases diameter subgroup. In the >1 cm in diameter metastasis sub-group analyses, LC differences were more pronounced with a 12-month LC of 71% in HFSRT vs. 47.7% in SRS (*p* = 0.003). Additionally, a higher BED was strongly associated with improved LC in univariate analysis (HR 0.94 per Gy, *p* = 0.002). Very interestingly, in melanoma histology only, LC was much improved in HFSRT with a 12-month LC of 59.8% vs. 46.6% in SRS group, *p* = 0.069. This is the first study to clearly identify the interest of HFSRT not only in large but also in small brain metastases. A single-center retrospective study [97] comparing different HFSRT schedules on a CyberKnife did not show any difference in the efficacy of 35 or 30 Gy in 5 fractions and 27 Gy in 3 fractions in 389 patients. In a dose-escalation study [98] of 46 patients regarding only large lesions (>3 cm diameter), the authors found a significant decrease in LC with a schedule of 24 Gy in 3 fractions vs. 27 or 30 Gy (12-month local progression free survival rate of 65%, 80%, 75% respectively). While no increase in LC was observed with the 10-Gy group, a higher rate of RN was reported (37% vs. 13% for the 9-Gy group and 0% for the 8-Gy group).

In a recent meta-analysis [21] of 15 studies that evaluated SRS vs. HFSRT schedules in patients with 1157 large brain metastases (>2 cm in diameter), no statistically significant difference but a trend in favor of HFSRT was found between the two groups with a 1-year local control of 76.7% for SRS vs. 83.1% for HFSRT (*p* = 0.38). This trend was increased in the 2 to 3 cm diameter group with a 12-month LC rate of 77.1% vs. 92.9% (*p* = 0.18) for SRS and HFSRT, respectively, while significant reduction of radionecrosis was observed (see hereafter in the dose–toxicity section). There was also no difference in the volume groups between lesions of 2–3 cm vs. lesions >3 cm (*p* = 0.14).

In addition, Shuryak et al. [99] reported that the LQ (Linear Quadratic) model, assuming the same tumoricidal mechanisms at all doses and fraction numbers, was a good predictor of the SRT tumor control probability at all dose ranges. Multifractionated regimens were slightly more effective than a single dose in SRT of brain metastases (937 patients), probably due to tumor reoxygenation between fractions.

While the combination of SRT with immunotherapy as an associated systemic treatment is becoming more common [100,101] and is showing promise [102,103,104] with good efficacy and tolerance, many unknowns remain on the dose administration schedules, especially in terms of fractionation. This combination has a potential superiority in using hypofractionated schedules for the induction of a pro-inflammatory response. In addition to its direct effects, it also has an effect on the microenvironment by inducing leukocyte adhesion molecules on vascular endothelial cells, increasing vascular permeability, and stimulating the expression of chemokines that facilitate T cell infiltration [105,106].

However, transposing in vitro in vivo results—which sometimes give opposite results—is not without difficulty, as Demaria et al. [107,108] and Lee et al. [109] have shown in pre-clinical studies. Notably, Dewan et al. [88] found in a pre-clinical study that fractionation combined with a CTLA-4 inhibitor through a putatively different molecular response induced an abscopal effect compared to single-dose irradiation with fractionation schedules close to those used in brain SRT (3 × 8 Gy, 5 × 6 Gy vs. 1 × 20 Gy). Similar results have been reported by Schaue et al. [110] and more recently by Vanpouille-Box et al. [89], who found an optimal synergy regarding efficacy by using an anti-CTLA-4 with a 3 × 8 Gy regimen compared to a single dose of 20 or 30 Gy. They found that the DNA exonuclease Trex1 was induced by doses above 12–18 Gy in cancer cells and attenuated their immunogenicity. Therefore, a repeated irradiation at a lower dose like 8 Gy is essential to stimulate the anti-tumor activity of immune cells [111]. Radiation schedules and tumor type are determinant in immunomodulatory potential, particularly in T cells responses [112]. Future research should investigate in prospective trial fractionated schedules with concurrent administration of immunotherapy and in each particular histology.

### 4.3. Dose–Toxicity Relation

Radionecrosis is the main complication of SRT and poses diagnostic difficulties. Overall rates vary from 0 to 12% of patients depending on the series and the definition (radiologic, metabolic, symptomatic) [26] but can reach 24% [113]. The RTOG 90–05 series reported an 11% rate of radionecrosis at 2 years. With the dose itself, the volume of tissue irradiated at a given dose is associated with greater toxicity. Therefore, a multi-fractionation schedule rather than a single dose allows the treatment of larger lesions since it limits the potential toxicity by decreasing the BED with a low α/β coefficient, according to the linear-quadratic model.

The first series to demonstrate the role of the volume of normal tissue irradiated in radionecrosis were those in which benign intracerebral tumors were treated by radiosurgery [114,115,116,117,118,119,120,121,122]. Among the brain metastasis exclusively series treated by SRT, Blonigen et al. [123] were the first to show that the identification of volumes receiving at least 10 and 12 Gy ($V_{10}$ and $V_{12}$) was predictive for radionecrosis in patients treated with LINAC-SRS. In their series of 63 patients with 173 lesions, the $V_{8\;},V_{10},\;V_{12},\;V_{14},\;V_{16}$ and the $V_{18}$ were significant predictive factors of radionecrosis in multivariate analysis for symptomatic and asymptomatic radionecrosis (except for the $V_{16}$ and $V_{18}$ for the latter), *p* < 0.0001. The more the volume increased, the greater the risk, with a linear increase. The authors concluded that hypofractionation should be considered if the $V_{10}$ is >10.5 cc or the $V_{12}$ > 8 cc, when the risk of radionecrosis is exceeded by about 20%. Minniti et al. [113] also showed that the ${\;V}_{10}$ and the $V_{12}$ were predictive of radionecrosis in a series of 206 patients with 310 lesions (*p* = 0.0001) treated with LINAC-SRS. Irradiation doses were 20 Gy at the margin for lesions <2 cm in diameter (4.3 cc), from 18 Gy for those between 4.3 cc and 14.1 cc and 15–16 Gy beyond that. For a $V_{10}$ > 12.6 cc and a $V_{12}$ > 10.9 cc, the risk of radionecrosis was 47%. The authors proposed using a hypofractionation for a $V_{12}$ > 8.5 cc, which corresponded to a 10% risk of radionecrosis. More recently, the same authors [17] again showed that the $V_{12}$ is the most significant variable of radionecrosis in the SRS treatment group with an incidence of 13% for a $V_{12}$ < 13.2 cc and 28% for a $V_{12}$ > 13.2 cc (*p* = 0.02). Other authors [116,124,125,126] also found $V_{10}$, $V_{12}$, $V_{14}$ to be significant factors with varying proportions of radionecrosis (see Table 2). An interesting study [127] showed the location of lesion as a predictive factor of RN in addition to $V_{12}\;\mathrm{and}\;V_{22}$.

For hypofractionated treatments, few data are available owing to the heterogeneity of the dose regimens administered. In the previous retrospective comparative study by Minniti et al. with a tri-fractionated regimen (3 × 9 Gy on the margin), they reported that $V_{18}$ was the most significant prognostic factor for radionecrosis with an incidence of 5% for a $V_{18}$ < 30.2 cc and 14% for a $V_{18}$ > 30.2 cc (*p* = 0.04). The increase in risk was progressive with size, with a risk of radionecrosis at 1 year of 0, 6, 13, 24% for $V_{18}$ < 22.8 cc, 22.8–30.2 cc, 30.3–41.2 cc and >41.2 cc, respectively. These results were similar to another series they had reported [81], identifying $V_{18}$ and $V_{21}$ as independent prognostic factors (*p* = 0.03). The risk of radionecrosis at 1 year was 14% for $V_{21}$ ≥ 20.9 cc and $V_{18}$ ≥ 26.2 cc and 4% for $V_{21}$ < 20.9 cc and $V_{18}$ < 26.2 cc, respectively. Inoue et al. [128] reported $V_{23.1}$ (excluding the GTV, equivalent to $V_{14}$ in single dose) as a predictive factor of radionecrosis using a 3-fraction schedule, with 3 cases of symptomatic radionecrosis requiring surgery above 7 cc, and a risk almost nil if < 5 cc. In a 5-fraction schedule, the same authors [129] reported a significant risk of symptomatic radionecrosis requiring surgery (2 cases) if the $V_{28.8}$ (excluding GTV) was >7 cc, none below and no edema requiring corticosteroids below 3 cc. Faruqi et al. [130] reported a limit of 10.5 cc for brain volume minus GTV receiving 30 Gy in a 5-fraction schedule, with 13% of <10.5 cc targets developing symptomatic radionecrosis vs. 61% if higher at 1 year. This study included surgical beds and in-place lesions. An interesting dosimetric study [131] reported that “hot spots” (105, 110, 111% and absolute dose of 33.5 Gy) between CTV and PTV (2 mm margin) were associated with more radionecrosis in 55 surgical beds treated in 5 fractions (25–35 Gy). Furthermore, a recent study [132] showed that $V_{25}$ and $V_{30}$ (brain minus GTV) were predictive of symptomatic radionecrosis, with 21% of cases at 2 years if both factors were $V_{25}$ > 16 cc and $V_{30}$ > 10 cc vs. 2% if both factors were below these thresholds.

Regarding retrospective HFSRT vs. SRS comparative studies, rates of radionecrosis or complications are lower in almost all HFSRT groups for similar or higher efficacy despite higher irradiation volumes (see Table 1). One study [96] identified a strong association of RN risk with ${\mathrm{BED}}_{2}$ in univariate analysis (HR 1.02 per Gy, *p* = 0.035). More interestingly, for lesions <1 cm in diameter, 12-month RN rate was 0% in HFSRT group vs. 9.6% for SRS group; the differences were more pronounced for metastases ≥1 cm with a RN rate of 3.9% vs. 21.2% for each group, respectively.

These results are similar to the radionecrosis rates observed in other retrospective HFSRT series, ranging from 0 to 15% [19,50,55,58,81,133]. In a dose-escalation [98] study of 46 patients regarding only large lesions (>3 cm diameter), the RN rate was associated with the dose delivered. They found a rate of RN of 0%, 13% and 37% for the 8-Gy, 9-Gy and 10-Gy in 3 fractions group respectively, while no increase in LC was observed with the 10-Gy group vs. 9-Gy group.

In the meta-analysis by Lehrer et al. [21], there was more radionecrosis in the SRS groups with an incidence of 18.2% vs. 7.1% for the HFSRT group (*p* = 0.02). More recently, Milano et al. [134] identified in a pooled published reports analysis that for brain metastasis brain plus target volume $V_{20}$ (3-fraction) or $V_{24}$ (5-fraction) < 20 cc was associated with <10% risk of any necrosis or edema, and <4% risk of radionecrosis requiring surgery. For three-fraction SRT for brain metastases, normal brain tissue $V_{18}$ < 30 cc and $V_{23}$ < 7 cc were associated with <10% risk of radionecrosis. Unlike these authors, we decided to present raw data of each particular study because the mathematical logistic models and NTCP modeling produce uncertainties in the data compilation, according to the authors themselves.

Other factors involved in the occurrence of radionecrosis have been reported, either related to the patient (sex, previous irradiation, location, chemotherapy [115,127,135,136] or to the administration of treatment (homogeneity, conformity index [117,137]). Overall, however, toxicities and particularly radionecrosis do not seem to be related to the treatment device used [22,23,28,33], although their dosimetric properties may differ. Nonetheless, a recent multi-institutional study [29] of 2699 lesions reported more radionecrosis in those treated with GK-SRS vs. LINAC-SRS after applying a propensity score (HR 4.42, *p* = 0.019). One study [137] showed that the compliance index was associated with more grade II complications or higher (*p* = 0.001). Regarding concurrent immunotherapy, the available studies may reassure us [101,138,139,140] but cautious should remain [141]. Only phase I/II trials are ongoing [142,143,144,145]. Future prospective data about fractionation schedules and concurrent delivery of SRT with immunotherapy are still needed.

## 5. Role of Tumor Volume

The role of tumor volume in the efficacy of stereotactic brain irradiation is difficult to interpret. A lower biological effective dose is generally used for large volumes, especially for single-fraction treatments. Fractionation might therefore offer greater efficacy thanks to the increase in the BED. In the RTOG 90-05 trial, multivariate analysis did not show a relationship between tumor volume, lesion diameter and local control. Tumor volume was also not shown to be a prognostic factor in multivariate analysis in the study by Shiau et al. [45], whereas the dose received was. Using the RTOG 90-05 dose regimens, Vogelbaum et al. [51] showed that local control was poorer in larger tumors, with a 6-month local control of 92, 87 and 71% for median tumor diameters of 1, 2.4 and 3.3 cm. However, the doses used were lower with increasing size according to the RTOG 90-05 dose regimen (15, 18 and 24 Gy). Local control at 1 year was similar: 45, 49, 85%, respectively. Chao et al. [53] reported lower local control rates at 6 and 12 months for lesions between 2 and 4 cm compared to 0 to 2 cm (83 and 62% vs. 97 and 92%, respectively, *p* < 0.0001) but BEDs were lower for larger lesions (between 28.6 and 36 Gy vs. 45.9–50.7 Gy) and the authors found that metastases receiving a dose >22 Gy were better controlled at 1 year than those receiving a lower dose (92 vs. 72%, *p* = 0.0013). Similar results were obtained by Molenaar et al. [54], with control improving with increasing dose, which was inversely correlated with lesion size. Furthermore, multivariate analysis showed that only lower doses decreased local control compared to higher doses (*p* = 0.007), not lesion size. In the series of Kwon et al. [79] mainly associating whole-brain irradiation and HFSRT, only volume emerged in univariate analysis and smaller lesions also received more doses compared to larger ones, as in the studies by Schomas et al. [146]. and Minniti et al. [81]. Tumor volume also did not emerge independently in older SRS series [4,147].

In series where only large lesions were treated, local control rates were broadly similar between studies compared to lesions of smaller volumes treated at a similar BED. The series of Higuchi et al. [19] had a mean lesion volume of 17.6 cc (10.8–35.5, between 3 and 4.5 cm in diameter) with a local control at 12 months of 76%, a rate comparable to series with much lower volumes (around 6–7 cc) in HFSRT [50,58,68]. The finding was similar in the series by Feuvret et al. [82], with a 100% local control at 12 months in the 12 patients treated by HFSRT with a median volume of 29.4 cc (equivalent diameter of 4.5 cm) and 58% in the 24 patients treated by SRS with a median volume of 15.69 cc (diameter 3.8 cm); Jiang et al. [148], with a 12-month local control of 94.2% for a median diameter of 4.1 cm (36 cc); and Wegner et al. [149], with a 1-year local control of 63% for a median volume of 15.6 cc. These series did not show any influence of volume on local control. Among the comparative retrospective studies, three [18,94,97] found volume to be pejorative, but for some of them, the efficacy was same or even better with larger lesions.

However, some studies have shown an effect of volume on efficacy with varying degrees of strength. The study by Murai et al. [150] showed that in patients with tumors >2.5 cm in diameter, local control at 6 and 12 months was 95% and 93% for volumes < 8 cc, 86% and 76% for between 8 and 15 cc, 80% and 67% for between 15 and 33 cc and 54% and 46% for >33 cc, *p* = 0.007. The 61 metastases were treated in HFSRT by CyberKnife in a relatively homogeneous manner. Matsuyama et al. [83] reported that tumor diameter was a pejorative predictive factor of local control in multivariate analysis (*p* = 0.001) in 334 patients for 497 lesions analyzed (8.6 mm median diameter (2.8–47.4)) treated with HFSRT at an effective biological dose of approximately 80 Gy (α/β = 10), regardless of size and with variable fractionation. Chang et al. [47] showed that brain metastases <1 cm treated with LINAC-SRS had better local control at 1 and 2 years compared to those >1 cm (0.5 cc); 86% and 78% vs. 56% and 24%, respectively (*p* = 0.0016). Lucia et al. [37] reported better local control in 134 patients with 208 lesions for those <2.04 cc vs. above (*p* < 0.0001, HR = 6.77). Becker et al. [84] showed that metastasis size ≤12 mm was a factor of better local control (*p* = 0.002) in 55 patients with 72 lesions on LINAC-SRS with doses ranging from 8 to 20 Gy (median 15 Gy). A retrospective monocenter study [151] involving 48 patients with 77 metastases treated homogeneously with LINAC-SRS at 20 Gy found that a GTV <2 cc was a factor of better local control (*p* = 0.037). Aoyama et al. [58] reported that a tumor volume <3 cc or >3 cc was significantly related in multivariate analysis to local control at 1 year with a rate of 96% vs. 59%, respectively (*p* = 0.0023). The median volume was 3.3 cc (approximately 1.8 cm in diameter) ranging from 0.006 to 48.3 cc. For Varlotto et al. [152], local control at 1 year was 95.2% vs. 83.3% for tumors <2 or >2 cc respectively, *p* = 0.0029. A study [153] evaluating the reduction in tumor volume at 2 months post SRS showed that a reduction in volume >50% was associated in multivariate analysis with a tumor volume <16 cc, *p* = 0.066 (the authors considered *p* < 0.1 to be significant). Similarly, some series showed that volume was an independent pejorative factor, although dose schedules, which varied widely and were generally inversely correlated with volume, were not described [30,154,155,156]. The comparative retrospective study by Ishihara et al. [18] showed that PTV volume was a prognostic factor in multivariate analysis, with a local control at 1 year of 94.4% vs. 77.5% for PTV <4 or >4 cc (*p* = 0.038), respectively. However, smaller tumors were treated more by SRS which has a higher BED, and no details on this were presented. Two other comparative studies, Mengue et al. [97]. and Lesueur et al. [94], found that high tumor volume was a poor predictor of local control in multivariate analysis. 

In total, 7 series out of 35 were globally homogeneous in their treatment and highlighted the pejorative role of volume. The main conclusion is that a higher dose is probably necessary when the volume increases but cautious should remain [98].

## 6. Conclusions

Stereotactic cerebral radiotherapy is the treatment of choice for patients with few or asymptomatic brain metastases. Considerable technical progress in recent years has made it possible to make wider, more comfortable and safer the use of this radiation modality, both for the practitioner and patients. The effects of the combination of SRT and systemic treatments such as targeted therapies or immunotherapy remain elusive, but the radiation schedule is a critical factor in the immunomodulatory responses. In this view, fractionated schedules may be preferred. Prospective trials on concurrent delivery of SRT with immunotherapy are still needed. In addition, fractionated schedules allow delivery of higher BED while preserving normal brain tissue, it may be considered not only in large metastases but from 1 cm diameter brain metastases as suggested by a retrospective study. Despite different dosimetric properties, no treatment device has been shown to be clearly more effective than another. While tumor volume is widely considered to be a pejorative prognostic factor in terms of efficacy, it can be assumed that a higher volume requires an increase in the effective dose. The volume of healthy tissue irradiated is a clear prognostic factor for radionecrosis but data are very heterogeneous and scarce, especially for fractionated schedules. With almost only retrospective data available, a 12-month local control rate >70% seems to be achieved with a ${\mathrm{BED}}_{10}$ of 40 to 50 Gy. A ${\mathrm{BED}}_{10}$ of 50 to 60 Gy seems to achieve a 12-month local control rate at least of 80% at 12 months. In the brain metastases radiosurgery series, for single-fraction schedule, a V12 Gy < 5 to 10 cc is associated to 7.1–22.5% radionecrosis rate. For the three-fraction schedule, V18 Gy < 26–30 cc, V21 Gy < 21 cc and V23 < 5–7 cc are associated to about 0–14% RN rate. For the five-fraction schedule, V30 < 10–30 cc, V28.8 Gy < 3–7 cc and V25 Gy < 16 cc are associated with about 2–14% symptomatic RN rate. We attempted with plenty of cautious to summarize the data presented here in Table 3 as a guide while waiting more robust studies. There are still no prospective trials comparing radiosurgery to multifractionated stereotactic irradiation. A phase III trial is ongoing for resected brain metastases [157].

## Figures and Tables

**Table 1:** Comparative retrospective studies of single-fraction (grey lines) vs. multi-fraction (white lines), local control (LC), ${\mathrm{BED}}_{10}$, ${\mathrm{BED}}_{3}$, radionecrosis and toxicities.

Authors	N. Patients	N. Metastases	Median Volume (PTV)	Doses/Prescription Isodoses	*GTV-PTV* Margin (mm)	Treatment Devices	Histology	Previous WBRT (%)	$BED_{10}$ (Gy)	LC at 6 Months (%)	LC at 12 Months (%)	$BED_{3}$ (Gy)	Radionecrosis (N)/Toxicities (%) (c)
Kim et al. (2011)	58	81	2.21 cc (0.03–24.34)	20 Gy (15–22)/90%	1	*LINAC*	Various	21%	60	81	71	153	1/17%
	40	49	5.00 cc (0.14–37.80)	36 Gy in 6 fr. (30–42)/91%	1			40%	57.6	97	69	108	0/5%
Wiggenraad et al. (2012)	41	46	<13 cc = 11 13–20 c = 17 >20 cc = 18	15 Gy	2	*LINAC*	Various	25%	37.5	89	67	90	NA
	51	65	<13 cc = 18 13–22 c = 16 >20 cc = 31	24 Gy in 3 fr./80%	2			15%	43.2	92	75	88	NA
Fokas et al. (2012)	107	NA	1.87 cc (0.03–11.17)	RTOG 90-05 schedule/NA	2	*LINAC*	Various	No	60	84	73	153	4/14%
	54 53		2.04 cc (1.17–18.71) 5.93 cc (2.7–23.16)	35 Gy in 7 fr./NA 40 Gy in 10 fr./NA	3 3				52.5 56	87 81	75 71	93.3 92	1/6% 0/2%
Feuvret et al. (2014)	24	24	18.31 cc (6.31–39.21)	14 Gy/70%	1	*LINAC*	Various	No	33.6	68	58	79.3	0/28%
	12	12	32.61 cc (19.10–65.56)	23.1 Gy in 3 fr./70%	2				41	100	100	82.4	0/25%
Ishihara et al. (2016)	53 in total	138	0.7 cc (0.1–8.3)	20 Gy (15–25)/80–90%	1	*LINAC*	Lung	7.6% In total	60	NA	NA (b)	153.3	21/0% (≥grade II)
		76	6.2 cc (0.1–29.5)	28 Gy (19.2–39) in 3–13 fr./80–90%	1–2				43.7 (a)	NA	83.6	80.3 (b)	5/0% (≥grade II)
Minniti et al. (2016)	151	179	12.2 cc (4.4–32)	18 Gy or 15–16 Gy/80–90%	1–2	*LINAC*	Various	No	50.4 or 37.5–41.6	94	77	126 or 90–101.3	31/NA
	138	164	17.9 cc (5.6–54)	27 Gy in 3 fr./80–90%	1–2				51.3	97	90	108	11/NA
Lesueur et al. (2018)	60 in total	52	0.47 cc	20 Gy (18–25)/80%	2	*CyberKnife*	Melanoma and kidney	No	60	89	79	153.3	5/NA
		141	1.75 cc	30 Gy (30–33) in 3–6 fractions/80%	2				60	80	72	90	10/NA
Chon et al. (2019)	67	154	9.7 cc (6.8–15.9)	20 Gy (18–22)/50%	0	*GammaKnife*	Various	No	60	92.9	66.6	153.3	NA/56%
	38	73	11 cc (6–14.8)	35 Gy (27–41) in 3 or 5 fractions/80%	0	*CyberKnife*			59.5 (5 fractions)	100	92.4	93.3	NA/36%
Loo et al. (2020)	152 in total	105	0.76 cc (0.27–11.4)	14 Gy/70%	2	*LINAC*	Various	No	33.6	94	88	79.3	1/NA
		141	4.51 cc (0.29–43.4)	23.1 Gy in 3 fr/70%	2				41	88	78	82.4	9/NA
Remick et al. (2020)	156 in total	222	0.7 cm (0.2–3.3) (*GTV*)	RTOG 90-05 schedule, median 24 Gy/50–80%	0/NA	*LINAC* *GammaKnife*	Various	33%	Median 81.6	NA	91	216	20/NA
		113	1.6 cm (0.2–5) (*GTV*)	Variable/2 to 5 fractions	NA	*LINAC*		34%	42.6	NA	85	-	8/NA
Putz et al. (2020)	120 in total	92	0.23 (0.12–0.50)	18 Gy (18–20)/80%	1–2	*LINAC*	Various	20.7%	50.4	>60%	55.6	126	8/NA
		98	1.42 (0.07–61.98)	40 Gy in 10 fr./80%	1–2			15.3%	56	>80%	70.2	93.3	2/NA

NA = not available, (a) BED calculation with marginal median dose to 5 fractions, (b) only available to <4 cc volume sub-group, (c) grade I to V according to CTCAE.

**Table 2:** Studies on brain metastases SRT reporting the normal brain volume as predictive factor of radionecrosis.

Authors (Year)	Treatment Machine	Number of Targets (Type)	Median Dose/ Number of Fractions	Definition of Predictive Factor	Predictive Dosimetric Factor	Radionecrosis Rate Above/Below of the Limit	Median Follow-Up (Months)
SINGLE FRACTION							
Peng (2019)	*CK*	294 (brain metastases)	20 Gy/1 fr. (14 to 25 Gy/1 to 5 fr.)	Brain + target	V14 ≥ 20 cc	12.1%/NR (all grades) 3.4%/NR (grade 3)	21.7
Minniti (2016)	*LINAC*	343 (brain metastases)	15 to 18 Gy	Brain − GTV	V12 > 13.2 cc	28%/13%	29
Sneed (2015)	*GK*	2200 (brain metastases)	19 Gy (7.5 to 20 Gy)	Brain + target	V12 > 3.3 cc V10 > 4.3 cc	13–14%/NR	9.9
Minniti (2011)	*LINAC*	310 (brain metastases)	18 Gy (15 to 20 Gy)	Brain − GTV	V10 > 12.6 cc V12 > 10.9 cc	47%/NR	9.4
Blonigen (2010)	*LINAC*	173 (brain metastases)	18 Gy (12 to 22 Gy)	Brain + target	V10 > 6.4 cc V12 > 4.8 cc	>34.6%/7.1%	13.7
Chin (2001)	*GK*	243 patients (variable lesions)	NR	Brain + target	V10 > 10 cc	76%/41%	NR
Korytko (2006)	*GK*	198 (variable lesions)	17.3 Gy (11–25)	Brain + target	V12 > 10 cc	55.3%/22.5% (symptomatic RN)	NR
Voges (1996)	*LINAC*	135 (variable lesions)	15 Gy (7 to 25 Gy)	Brain + target	V10 > 10 cc	23.7%/0%	28.1
THREE FRACTIONS							
Dore (2017)	*LINAC*	97 (surgical cavities)	23.1 Gy/3 fr.	Brain − PTV (2 mm margin CTV-PTV)	V21 (median 4.6 cc in cohort without RN vs. 10 cc in cohort with RN)	NR	17
Minniti (2016)	*LINAC*	343 (brain metastases)	27 Gy/3 fr.	Brain − GTV	V18 > 30.2 cc	14%/5%	29
Minniti (2014)	*LINAC*	171 (brain metastases)	27 Gy/3 fr. (27 to 36 Gy/3 fr.)	Brain − GTV	V21 ≥ 20.9 cc V18 ≥ 26.2 cc	14%/4%	11.4
Inoue (2013)	*CK*	159 (brain metastases)	27 Gy/3 fr.	Brain − GTV	V23.1 ≥ 5 cc V23.1 ≥ 7 cc	5.7%/1.4% 9%/0% (RN requiring surgery)	7
Minniti (2013)	*LINAC*	101 (surgical cavities)	27 Gy/3 fr.	Brain − GTV	V24 ≥ 16.8 cc	16%/2%	16
FIVE FRACTIONS							
Andruska (2020)	*GK* *LINAC*	117 (brain metastases + surgical cavities)	30 Gy/5 fr. (25 to 35 Gy/5 fr.)	Brain − GTV	V25 > 16 cc V30 > 30 cc	21%/2% (symptomatic RN)	10.3
Faruqi (2020)	*LINAC*	250 (brain metastases + surgical cavities)	30 Gy/5 fr. (20 to 35 Gy/5 fr.)	Brain − GTV/CTV	V30 ≥ 10.5 cc	61.1%/12.6% (symptomatic RN)	12
Inoue (2014)	*CK*	85 (brain metastases)	31 Gy/5 fr. (25 to 40 Gy/5 fr.)	Brain − GTV	V28.8 > 7 cc V28.8 > 3 cc	12.5%/0% (RN requiring surgery) 11.2%/0% (symptomatic RN)	8
Ernst-Stecken (2006)	*LINAC*	72 (brain metastases)	30–35 Gy/5 fr.	Brain − GTV	V20 > 23 cc	70%/14%	7

Vx: volume of receiving x Gy. NR = not reported, CK = CyberKnife, LINAC = Linear accelerator, GK = GammaKnife, RN = radionecrosis.

**Table 3:** Summary of dose-efficacy and dose-toxicity data in brain metastasis SRT studies.

**DOSE—EFFICACY DATA**				
BED	Expected 12-months LC	Evidence level	Comments	Perspectives/future research
${\mathrm{BED}}_{10}$ 40 to 50 Gy ${\mathrm{BED}}_{10}$ 50 to 60 Gy	>70% >80%	Moderate	Consider fractionation if: - concurrent IT, - lesion > 2 cm in diameter (${\mathrm{BED}}_{10}$ of 50 Gy is corresponding to about 18 Gy in 1 fr., 27 Gy in 3 fr. or 30 Gy in 5 fr.)	Single fraction vs. multiple fractions in phase III trials Concurrent IT/TKI Specific histology data
**VOLUME OF BRAIN CONSTRAINTS**				
Vx	Expected RN rate	Evidence level	Brain structure for the constraints	Perspectives/future research
Single fraction V12 Gy < 5 to 10 cc	7.1 to 22.5%	Moderate	Whole brain (including target volumes) in almost studies but consider Brain—GTV	Single fraction vs. multiple fractions in phase III trials Concurrent IT/TKI Specific histology data
Three fractions V18 Gy < 26–30 cc V21 Gy < 21cc V23 Gy < 5–7 cc	0 to 14%	Weak	Brain − GTV in 4 studies out 5	
Five fractions V30 Gy < 10–30 cc V28.8 Gy < 3–7 cc V25 Gy < 16 cc	2 to 14%	Weak	Brain − GTV/CTV (rate of symptomatic necrosis)	

${\mathrm{BED}}_{10}$: biological effective dose with alpha/beta ratio = 10 Gy. Vx: volume of x Gray. RN: radionecrosis. IT: immunotherapy. TKI: tyrosine-kinase inhibitor. GTV: gross tumor volume. CTV: clinical tumor volume.
